# Metformin alleviates breast cancer through targeting high‐mobility group AT‐hook 2

**DOI:** 10.1111/1759-7714.13318

**Published:** 2020-02-07

**Authors:** Yang Li, Dan Wang, Hui Ren, Wei Feng

**Affiliations:** ^1^ Department of Anesthesiology China‐Japan Union Hospital of Jilin University Changchun China; ^2^ Department of Breast Surgery The Second Hospital of Jilin University Changchun China; ^3^ Department of General Surgery China‐Japan Union Hospital of Jilin University Changchun China

**Keywords:** Breast cancer, growth, HMGA2, metformin

## Abstract

**Background:**

As a classic oral drug used in diabetic patients, metformin has exhibited an anticancer role in many types of cancers in recent years. Here, we aimed to investigate the role of metformin in the growth of breast cancer and its novel targets.

**Methods:**

A cell viability assay was utilized to examine the inhibitory effect of metformin on proliferation of breast cancer cells. Western blotting and RT‐PCR assays were used to detect the regulation of metformin on the expression of oncogenic HMGA2. The luciferase reporter vector of HMGA2 promoter was constructed. A luciferase reporter gene assay was performed to analyze the effect of metformin on HMGA2 promoter activity in breast cancer cells. Chromatin immunoprecipitation (ChIP) assay was performed to show the binding of Sp1 to HMGA2 promoter in breast cancer cells with or without metformin treatment. The function of metformin‐regulated HMGA2 in breast cancer growth was tested using a cell viability assay.

**Results:**

Cell proliferation was obviously inhibited in breast cancer cells treated with different concentrations of metformin. The level of mRNA and protein of HMGA2 was significantly reduced by metformin in the cells. Mechanistically, metformin was able to inactivate the HMGA2 promoter through downregulating transcription factor Sp1 in the cells. In terms of function, treatment with metformin suppressed the proliferation of breast cancer cells and overexpressed HMGA2 reversed the inhibition of cell proliferation mediated by metformin.

**Conclusion:**

Metformin resists the growth of breast cancer through targeting Sp1/HMGA2 signal.

## Introduction

Breast cancer is predominantly a malignant tumor in women, with high mortality rates worldwide.^1^ Serving as an oral hypoglycemic drug, metformin is used in the treatment of diabetes through AMPK signaling pathway.^2^ A previous investigation revealed a low incidence of cancer in diabetic patients treated with metformin.^3^ An increasing number of studies display the relationship among metformin treatment and cancer incidence or patient survival.^4^, ^5^, ^6^ Metformin is able to inactivate STAT3 and NF‐κB to suppress IL‐6‐induced breast cancer progression.^7^ Some researchers have observed that metformin can reduce the development of tumor and prolong the survival of patients with T2DM and different cancers, such as prostate cancer, lung cancer, colon cancer or breast cancer.^8^, ^9^ In certain types of cancer including melanoma, ovarian, prostate, lung, colon or breast cancers, metformin and phenformin have been shown to prevent tumor progression.^10^, ^11^, ^12^, ^13^, ^14^ Metformin together with phenformin also play roles in resisting the development of colon cancer through promoting AMPK and ROS production and inhibiting glycolysis.^15^, ^16^, ^17^ However, the underlying mechanism of metformin in cancer therapy needs to be further investigated.

As an architectural transcription factor, high‐mobility group AT‐hook 2 (HMGA2) binds to the AT‐rich regions in DNA through its three basic DNA‐binding domains called “AT‐hooks.”^18^ By changing chromatin structure, HMGA2 can regulate transcription to affect the expression of many mammalian genes.^18^ High HMGA2 is frequently found in tumor tissues but rarely in normal tissues.^18^, ^19^ Overexpressed HMGA2 is closely associated with poor survival of breast, colorectal or lung cancer patients.^20^, ^21^, ^22^ The evidence proves that oncogenic HMGA2 participates in DNA damage repair, stem cell self‐renewal, or tumor growth.^23^, ^24^, ^25^, ^26^ HMGA2 is considered to promote tumor development in part through its target genes. For instance, it has been reported that HMGA2 counteracted the transcription repressor p120^E4F^ to induce cyclin A expression, resulting in cell cycle progression.^27^ Human telomerase reverse transcriptase (hTERT) can be activated by HMGA2, holding back the telomere shortening in cancer cells.^28^ In addition, several studies report that HMGA2 was able to directly activate some pro‐metastatic genes, CXCR4, SNAIL or SLUG.^29^, ^30^, ^31^ Much attention has been focused on the regulatory cascades of HMGA2 expression during cancer progression. It has been shown to be activated by Wnt/β‐catenin signaling and repressed by the ZBRK1/BRCA1/CtIP pathway.^32^, ^33^ Yet, whether HMGA2 is involved in metformin‐associated breast cancer growth inhibition remains unclear.

In our study, we aimed to clarify the role of HMGA2 in metformin‐inhibited breast cancer and its underlying regulatory mechanism. Metformin is able to effectively stifle breast cancer growth in vitro. For the mechanism investigation, metformin inactivates oncogenic HMGA2 transcription through reducing transcription factor Sp1, leading to breast cancer growth inhibition. Our findings might provide another option for clinical breast cancer therapy.

## Methods

### Cell lines

Breast cancer cell line MCF‐7 was grown in RPMI Medium 1640 (Gibco, USA) containing 10% fetal calf serum (FCS, Gibco). Another breast cancer cell line BT‐474 was grown in DMEM medium (Gibco), 10% FCS, 100 U/mL penicillin, and 100 μg/mL streptomycin in humidified 5% CO_2_ at 37°C.

### Cell viability assay

MTT assay was applied to analyze the proliferation ability of breast cancer MCF‐7 and BT‐474 cells. Breast cancer cells (3000 cells/well) were plated on 96‐well plates with at least three replicates and then incubated for 10 hours to form confluent monolayers. After the cells were treated with metformin, 10 μL MTT (5 mg/mL) was added into each well and the absorbance values were evaluated at OD_490nm_ using the absorbance reader.

### RNA extraction and PCR

TRIzol reagent was used to extract total RNA from breast cancer MCF‐7 or BT‐474 cells and 1 μg RNA was reversely transcribed into cDNA for every sample. The mRNA levels of HMGA2 and GAPDH were detected through RT‐PCR assay by following the standard protocol. It should be noted that GAPDH served as the internal control for HMGA2 detection.

### Western blot analysis

Post‐treatment with different doses of metformin, total protein of MCF‐7 and BT‐474 cells were extracted by using RIPA lysis buffer. The same amount of total protein for each sample was running in SDS‐PAGE gel. The primary antibodies including anti‐HMGA2 and anti‐β‐actin were purchased from GeneTex Inc. (Irvine, USA) and Proteintech (Chicago, USA). The secondary antibodies including goat anti‐rabbit (Abcam, USA) or anti‐mouse antibody (Abcam) were incubated with western blots and the membrane was then visualized by ECL (Millipore). Image J software was used to quantify the intensity in western blotting analysis.

### Luciferase reporter gene assay

MCF‐7 cells (1 × 10^4^ cells/well) were seeded into 24‐well plates with ~70% confluence with at least three repeats for each experiment and then the cotransfection of pGL3‐HMGA2, pRL‐TK (Promega, USA) and/or metformin treatment (40 mM or 80 mM) were performed. pRL‐TK was applied for internal normalization. The cells were collected for the luciferase activity analysis 48 hours later. Luciferase activity was determined using Dual‐Luciferase Reporter Gene Assay System (Promega) following the manufacturer's instructions.

### Chromatin immunoprecipitation (ChIP) analysis

EpiQuik chromatin immunoprecipitation kit from Epigentek Group Inc. (Brooklyn, NY) was used in chromatin immunoprecipitation (ChIP) assay by following the manufacturer's instructions. The protein‐DNA complexes were immunoprecipitated with anti‐Sp1 or normal rabbit IgG as a negative control antibody. DNA from the above samples was then subjected to PCR assay.

### Statistical analysis

Each experiment was performed in triplicate. Statistical significance was evaluated by comparing (± SD) using a Student's *t*‐test. Nonsignificant difference was marked with ns. Criterion for statistically significant differences were as follows: ***P < 0.001; **P < 0.01; *P < 0.05.

## Results

### Metformin restrains the proliferation of breast cancer MCF‐7 and BT‐474 cells

**Figure 1:**
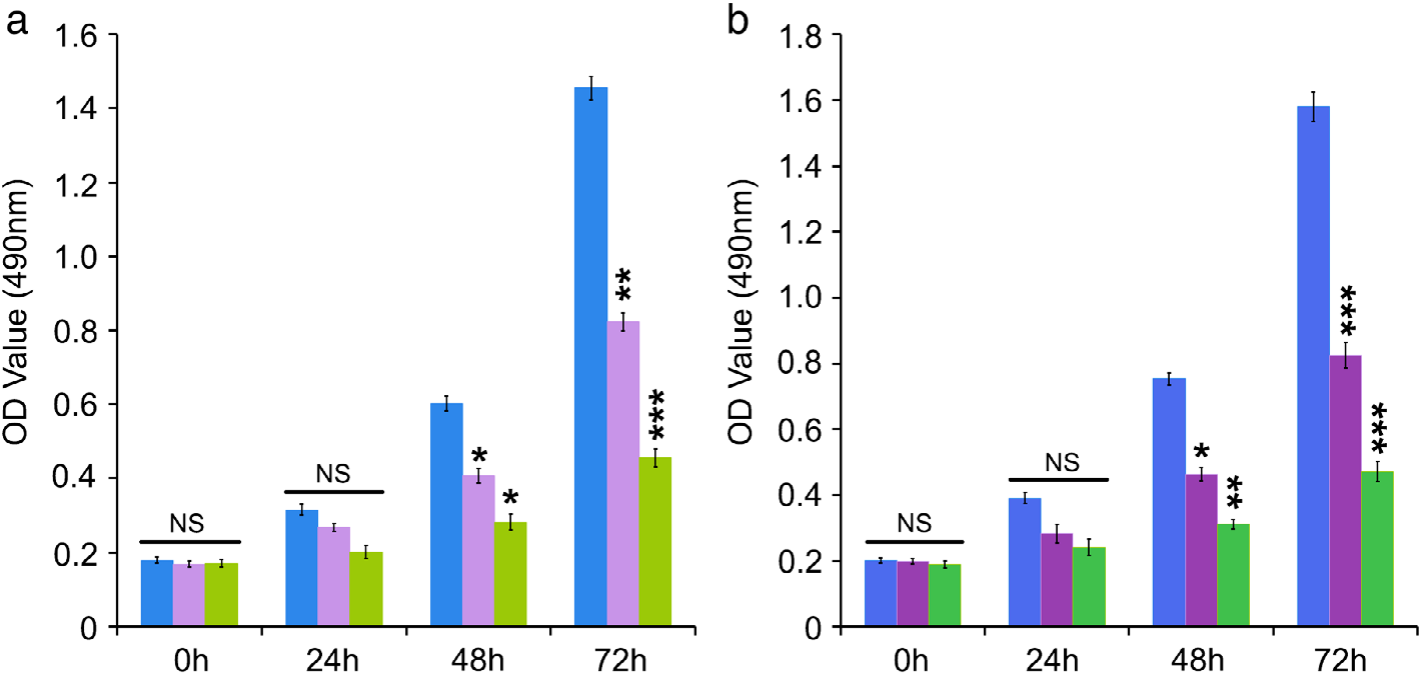
Metformin restrained the proliferation of breast cancer MCF‐7 and BT‐474 cells. (**a**, **b**) The effect of metformin (Met) treatment on the proliferation of MCF‐7 and BT‐474 cell lines was examined by MTT assay. ***P < 0.001; **P < 0.01; *P < 0.05; ns, not significant. (**a**) MCF‐7 (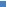) Met 0 mM, (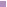) Met 40 mM and (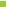) Met 80 mM. (**b**) BT‐474 (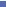) Met 0 mM, (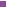) Met 40 mM and (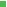) Met 80 mM.

As an oral drug controlling blood glucose in diabetes patients, metformin exerts an antiproliferative effect in multiple cancers including prostate cancer, lung cancer, colon cancer or breast cancer.^8^, ^9^ However, the function of metformin in preventing breast cancer growth and molecular regulatory mechanism is unclear and needs further clarification. We subsequently performed a cell viability analysis using breast cancer MCF‐7 and BT‐474 cell lines with or without the administration of metformin. Treatment with increased concentrations of metformin significantly reduced the cell viability of breast cancer (Fig 1a,b). These data indicate that metformin is capable of effectively preventing the proliferation of breast cancer cells.

### Metformin inhibits oncogenic HMGA2 in breast cancer

**Figure 2:**
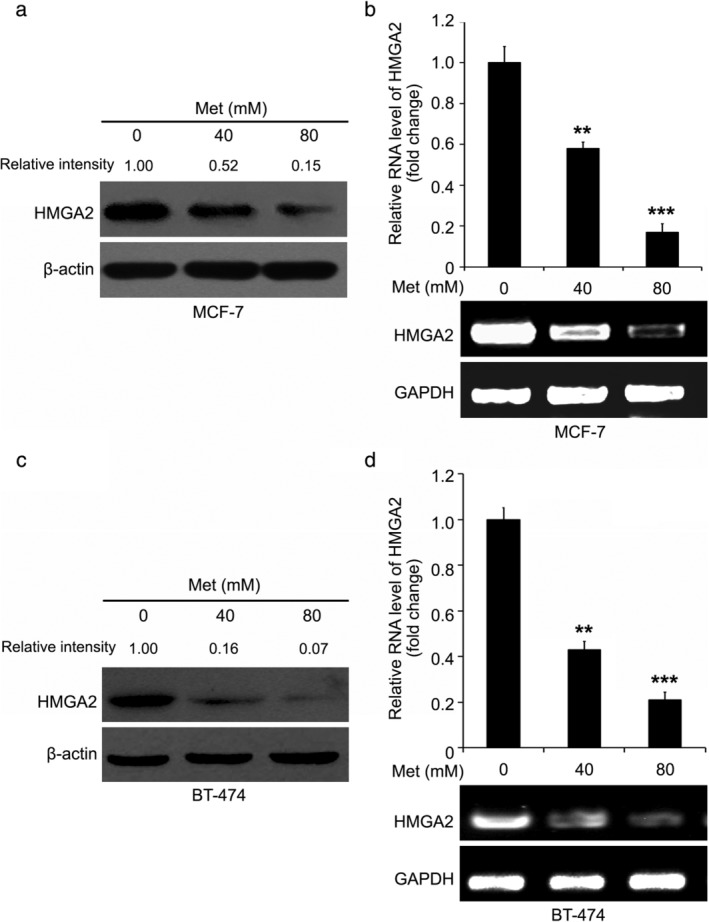
Metformin inhibited oncogenic HMGA2 in breast cancer. (**a**, **b**) Analysis of the level of mRNA and protein of HMGA2 in MCF‐7 cell line with the addition of metformin was detected by western blotting and RT‐PCR analysis. The density of the bands in western blotting analysis was quantified using ImageQuant software. The statistical change from three independent experiments for RT‐PCR assay is presented. Statistically significant differences in (**b**) were ***P < 0.001; **P < 0.01. (**c**, **d**) The level of mRNA and protein of HMGA2 in BT‐474 cell line with the addition of metformin was detected by western blotting and RT‐PCR analysis. The density of the bands in western blotting analysis was quantified using ImageQuant 5.2 software. Statistically significant differences in (**d**) were ***P < 0.001; **P < 0.01.

HMGA2, an architectural transcription factor, is abundant in tumor cells and its high expression is related to poor survival rates in many types of cancers including breast cancer, colorectal cancer or lung cancer.^18^, ^19^, ^20^, ^21^, ^22^ Yet, the role of HMGA2 in metformin‐mediated breast cancer inhibition is as yet undetermined. Here, we focus on whether HMGA2 takes part in metformin‐depressed breast cancer. After breast cancer MCF‐7 and BT‐474 cells were treated with an increased dose of metformin, the mRNA and protein level of HMGA2 was evaluated through western blotting and RT‐PCR assays. Compared to the control group, HMGA2 was obviously decreased by metformin at the protein level in MCF‐7 cell line (Fig 2a). Furthermore, we wanted to investigate whether metformin could modulate the HMGA2 transcription in the cells. We observed that the mRNA level of HMGA2 was markedly decreased by metformin in breast cancer cells (Fig 2b). We further confirmed the above data in another breast cancer cell line BT‐474 (Fig 2c,d). Thus, we found that HMGA2 could be reduced by metformin in breast cancer.

### Metformin targets transcription factor Sp1 to control HMGA2 transcription

**Figure 3:**
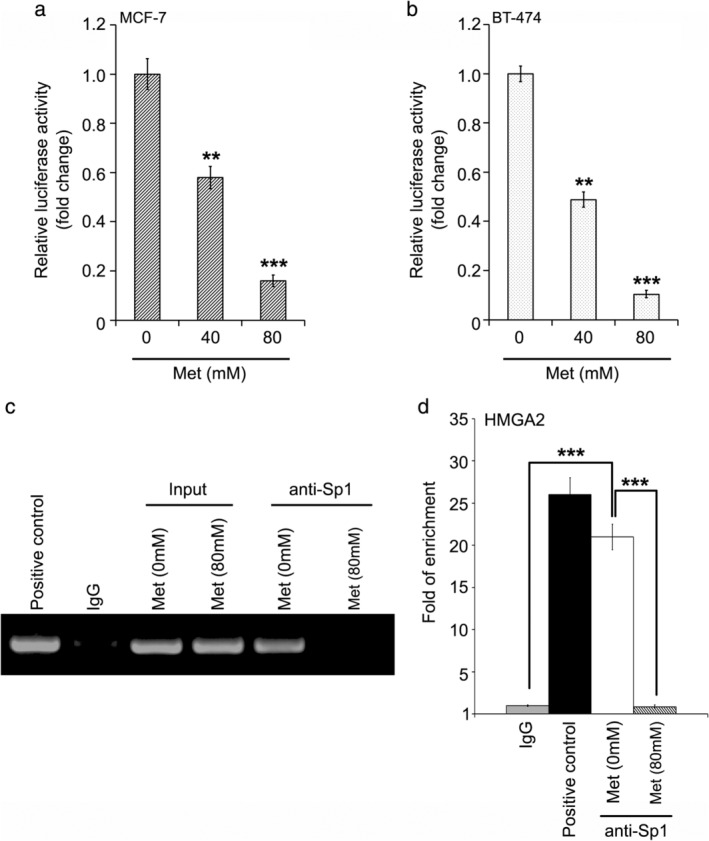
Metformin targeted transcription factor Sp1 to control HMGA2 transcription. (**a**, **b**) Effect of metformin (Met) on HMGA2 transcription in MCF‐7 and BT‐474 cell lines was analyzed by luciferase reporter gene assay. ***P < 0.001; **P < 0.01. (**c**, **d**) The binding of Sp1 to HMGA2 promoter with or without the addition of metformin was tested by ChIP and PCR or quantitative real‐time PCR assays.

Taking a step further, we then attempted to clarify how metformin regulates HMGA2 in breast cancer. According to the protocol used in a previous study,^34^ we cloned the core promoter region into pGL3‐basic plasmid. We then analyzed whether metformin could regulate the promoter activities of HMGA2 in breast cancer cells. Our data showed that metformin was able to significantly decrease the luciferase activities of pGL3‐HMGA2 promoter in MCF‐7 and BT‐474 cells (Fig 3a,b). As shown in a previous investigation^34^ and the prediction using the online bioinformatics tool JASPAR database (http://jaspar.binf.ku.dk/cgi-bin/jaspar_db.pl), transcription factors Sp1 had binding sites in the core promoter region of HMGA2. Accordingly, we tested whether metformin affected the binding of Sp1 to HMGA2 promoter through ChIP‐PCR and ChIP‐quantitative real‐time PCR assay. Our data showed that the binding of Sp1 to HMGA2 promoter was abrogated by metformin in breast cancer cells (Fig 3c,d). In summary, we concluded that metformin could control HMGA2 transcription through targeting Sp1 in breast cancer.

### HMGA2 is a mediator in metformin‐alleviated breast cancer

**Figure 4:**
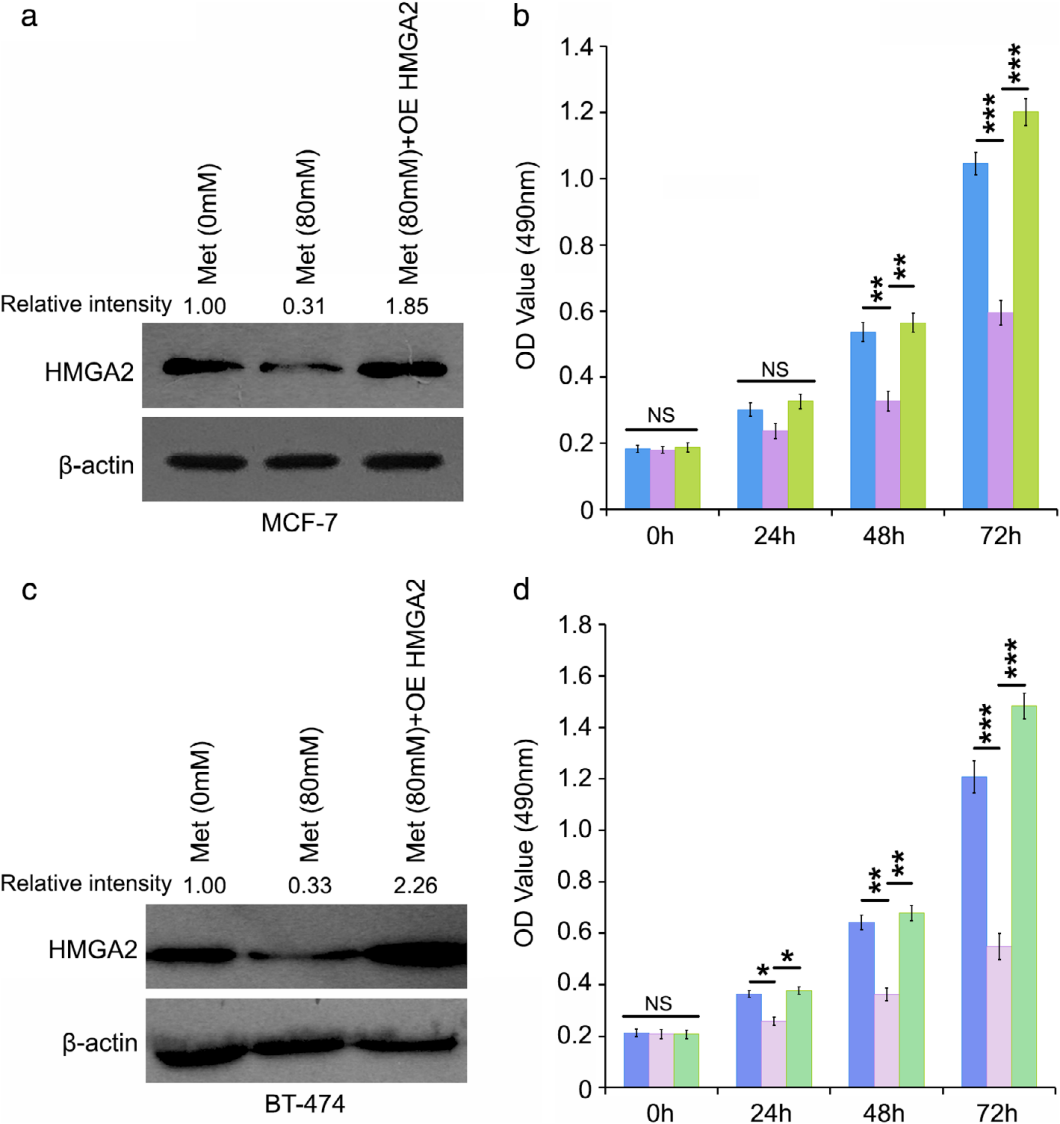
HMGA2 is a mediator in metformin‐alleviated breast cancer. (**a**, **c**) The level of MCF‐7 and BT‐474 cell lines with the addition of metformin and/or HMGA2 overexpression (OE HMGA2) was analyzed by western blotting. The density of the bands in the western blotting analysis was quantified using ImageQuant software. (**b**, **d**) MTT assay was applied to analyze the effect of metformin and/or HMGA2 overexpression (OE HMGA2) on the proliferation of MCF‐7 and BT‐474 cell lines. ***P < 0.001; **P < 0.01; *P < 0.05; ns, not significant. (**b**) MCF‐7 (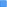) Met (0 mM), (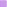) Met (80 mM) and (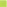) Met (80mM)+OE HMGA2. (**d**) BT‐474 (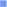) Met (0 mM), (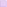) Met (80 mM) and (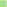) Met (80 mM)+OE HMGA2.

Based on the finding that metformin regulated HMGA2 in breast cancer cells, we finally analyzed the function of HMGA2 in metformin‐inhibited proliferation of breast cancer cells. First, we evaluated the level of HMGA2 in MCF‐7 or BT‐474 cells treated with metformin and/or HMGA2 transfection (Fig 4a,c). For the administration of 80 mM metformin, the proliferation of MCF‐7 or BT‐474 cells was obviously reduced. Moreover, HMGA2 overexpression could reverse the decrease in cell proliferation mediated by metformin (Fig 4b,d). Collectively, we concluded that metformin could alleviate breast cancer through targeting HMGA2.

## Discussion

Serving as an oral hypoglycemic drug, metformin is widely applied in clinical therapy. Metformin functions in diabetes treatment through AMPK signaling pathway.^2^ It has been reported that diabetic patients with metformin treatment had a low incidence of cancer.^3^ There exists a close correlation among metformin treatment and cancer incidence or patient survival.^4^, ^5^, ^6^ For the molecular mechanism of metformin in cancer treatment, it has been reported to inactivate STAT3 or NF‐κB, preventing IL‐6‐induced breast cancer progression.^7^ In prostate cancer, lung cancer, colon cancer or breast cancer patients with T2DM, metformin has been shown to reduce the development of tumor and prolong the survival.^8^, ^9^ Metformin together with phenformin also resists the development of colon cancer through promoting AMPK and ROS production and inhibiting glycolysis.^15^, ^16^, ^17^ In our study, we were interested in ascertaining the novel targets of metformin in cancer, especially in breast cancer.

First, we tried to evaluate the function of metformin in the development of breast cancer. An increased dose of metformin was able to markedly depress cell viability in breast cancer and confirms that metformin has the ability of suppressing the growth of breast cancer. As an oncogenic architectural transcription factor, HMGA2 can interact with the AT‐rich regions of DNA via its “AT‐hooks” domain.^18^ High HMGA2 is frequently observed in tumor tissues but nearly disappears in normal tissues.^18^, ^19^ HMGA2 augmentation is closely correlated with poor survival of breast, colorectal or lung cancer patients.^20^, ^21^, ^22^ The evidence proves that oncogenic HMGA2 participates in DNA damage repair, stem cell self‐renewal, or tumor growth.^23^, ^24^, ^25^, ^26^ HMGA2 is considered to promote tumor development in part through its target genes.

In this study, we were curious to ascertain the function of HMGA2 in metformin‐inhibited growth of breast cancer cells. The data showed that oncogenic HMGA2 could be sharply downregulated by metformin at the promoter, mRNA and protein level. We then attempted to clarify how metformin modulated oncogenic HMGA2. A previous study^34^ and website prediction demonstrated that transcription factor Sp1 might activate HMGA2 transcription, implying that Sp1 could take part in metformin‐downregulated HMGA2 in breast cancer cells. The results from the ChIP assay proved that the binding of Sp1 to HMGA2 promoter could be reduced by treatment with metformin. Finally, our data revealed that HMGA2 was involved in metformin‐suppressed breast cancer.

In summary, the findings from our study presents a novel mechanism for ameliorating breast cancer under metformin treatment. Metformin can effectively stifle breast cancer growth. As a novel target, HMGA2 is obviously decreased in metformin‐resisted breast cancer. Metformin regulates transcription factor Sp1 to reduce HMGA2 activation, leading to the inhibition of breast cancer growth. Our finding emphasizes that metformin should be used in breast cancer therapy.

## Disclosure

No authors report any conflict of interest.
